# Inverse Association of the Life’s Crucial 9 Score With MAFLD: An Exploratory Mediation Analysis of the C‐Reactive Protein–Albumin–Lymphocyte (CALLY) Index

**DOI:** 10.1155/ije/1340865

**Published:** 2026-10-17

**Authors:** Hanlu Li, Tianju Li

**Affiliations:** ^1^ Department of Infectious Diseases The Ninth People’s Hospital of Chongqing (Beibei Affiliated Hospital of Chongqing Medical University) Chongqing China

**Keywords:** CALLY index, Life’s Crucial 9, mediation analysis, metabolic dysfunction–associated fatty liver disease (MAFLD), NHANES

## Abstract

**Background:**

Metabolic dysfunction–associated fatty liver disease (MAFLD) is strongly associated with cardiovascular disease. Life’s Crucial 9 (LC9), an expanded cardiovascular health metric incorporating psychological health, may provide a multidimensional measure of cardiometabolic health. The CALLY index integrates inflammatory, immune, and nutritional information and has been associated with MAFLD. However, whether ln(CALLY) statistically accounts for part of the association between LC9 and MAFLD remains unclear. This study aimed to examine the association between LC9 and MAFLD and to explore the statistical indirect association through ln(CALLY).

**Methods:**

Data were obtained from the National Health and Nutrition Examination Survey (NHANES) conducted between 2005 and 2016. Weighted logistic regression, restricted cubic spline (RCS) modeling, subgroup analysis, and weighted quantile sum (WQS) regression were used to assess the relationship between LC9 and MAFLD. Exploratory mediation analysis was performed to evaluate the statistical indirect association through ln(CALLY). Sensitivity and supplementary analyses were conducted to assess the robustness of the findings with respect to missing data, alternative disease definitions, definitional overlap, alternative cardiovascular health metrics, individual LC9 components, and mediator choice.

**Results:**

This study included 3841 participants. Higher LC9 scores were significantly associated with lower odds of prevalent MAFLD. Specifically, every 10‐point increase in the LC9 score was associated with lower odds of MAFLD (OR = 0.51, 95% CI: 0.47–0.56, *p* < 0.001). Participants in the highest tertile of the LC9 score had lower odds of MAFLD (OR = 0.16, 95% CI: 0.12–0.22, *p* < 0.001) compared with those in the lowest tertile. Similarly, ln(CALLY) was inversely associated with MAFLD (OR = 0.57, 95% CI: 0.52–0.64, *p* < 0.001). Exploratory mediation analysis indicated that ln(CALLY) statistically accounted for approximately 17.7% of the LC9–MAFLD association (*p* < 0.001). Sensitivity and supplementary analyses showed that the association was attenuated but remained statistically significant after exclusion of overlapping metabolic components from LC9 (OR per 10‐point increase in modified LC9 = 0.891, 95% CI: 0.832–0.955, *p* = 0.002) and remained materially consistent when MASLD was used as an alternative outcome (OR = 0.545, 95% CI: 0.494–0.602, *p* < 0.001). No significant indirect association was observed using PNI as an alternative mediator (*p* = 0.988). WQS analysis assigned relatively larger weights to BMI, blood glucose, and psychological health.

**Conclusion:**

Higher LC9 scores were associated with lower odds of prevalent MAFLD. Exploratory mediation analysis suggested a statistical indirect association through ln(CALLY), although causal or temporal mediation cannot be inferred. Further prospective studies are needed to confirm these findings.

## 1. Introduction

Metabolic dysfunction–associated fatty liver disease (MAFLD), previously referred to as nonalcoholic fatty liver disease, was proposed to emphasize the central role of metabolic dysfunction in fatty liver disease and to improve risk stratification in clinical and epidemiological research [1]. As one of the most common chronic liver diseases worldwide [2], MAFLD affects a substantial proportion of adults and imposes a growing burden on healthcare systems [3]. In addition to liver‐related outcomes, MAFLD is closely linked to obesity, insulin resistance, type 2 diabetes, dyslipidemia, and hypertension, and cardiovascular disease remains a major cause of death in this population [4]. Given its high prevalence and broad systemic consequences, identifying modifiable factors associated with MAFLD is important for prevention and early intervention.

Cardiovascular health (CVH) metrics have increasingly been used to evaluate overall cardiometabolic health. The American Heart Association (AHA) first introduced Life’s Simple 7 (LS7) in 2010 and later updated it to Life’s Essential 8 (LE8) by adding sleep health [5, 6]. Recent research has suggested that psychological well‐being is also relevant to cardiometabolic outcomes. On this basis, Life’s Crucial 9 (LC9) has been proposed as an expanded CVH construct that incorporates depressive symptoms, commonly assessed using the Patient Health Questionnaire‐9 (PHQ‐9), in addition to the LE8 components [7]. Previous studies have shown that better CVH, as reflected by higher LS7 or LE8 scores, is associated with a lower likelihood of fatty liver disease [8, 9]. Moreover, depressive symptoms themselves have been linked to metabolic dysfunction and fatty liver [10, 11]. However, evidence regarding the relationship between LC9 and MAFLD remains limited.

Chronic low‐grade inflammation is considered an important mechanism in the development and progression of MAFLD [12]. Inflammatory and metabolic disturbances may alter liver function, nutritional status, and immune responses simultaneously, suggesting that composite biomarkers may better capture disease‐related systemic changes than single laboratory indicators alone [13, 14]. The C‐reactive protein–albumin–lymphocyte (CALLY) index, calculated from CRP, serum albumin, and lymphocyte count, has been proposed as an integrated marker reflecting inflammation, nutritional condition, and immune status [15]. Previous studies have associated the CALLY index with adverse outcomes in several chronic diseases and with metabolic abnormalities [16, 17]. However, whether a statistical indirect association through ln(CALLY) exists between LC9 and MAFLD remains unclear.

In the present study, we used nationally representative data to examine the association between LC9 and MAFLD and to explore the statistical indirect association through ln(CALLY). We further performed a series of sensitivity and supplementary analyses to assess the robustness of the findings across alternative analytical approaches and disease definitions.

## 2. Methods

### 2.1. Data Sources and Population

The National Health and Nutrition Examination Survey (NHANES) is an ongoing cross‐sectional survey designed to collect health‐related information from a nationally representative sample of the noninstitutionalized U.S. population. The study protocol was approved by the National Center for Health Statistics Institutional Review Board, and written informed consent was obtained from all participants. NHANES employs a multistage probability sampling design to ensure comprehensive and reliable data that reflect the health status of the U.S. population.

Data from 60,936 participants were obtained from NHANES cycles conducted between 2005 and 2016. The exclusion criteria were as follows: (1) individuals under 20 years of age (*n* = 26,756) and pregnant women(*n* = 513; (2) individuals with liver disease due to other causes (*n* = 1196); (3) excessive alcohol consumption (*n* = 5802); (4) missing data required to calculate the U.S. Fatty Liver Index (US‐FLI) and cardiometabolic factors (*n* = 10,528); (5) incomplete data for any of the nine LC9 components (*n* = 7572); (6) missing data required to calculate the CALLY index (*n* = 4174); and (7) incomplete covariate data required for the primary analyses (*n* = 554). Ultimately, 3841 eligible participants were included in the final analysis. The selection and exclusion process is illustrated in Figure 1.

**FIGURE 1:**
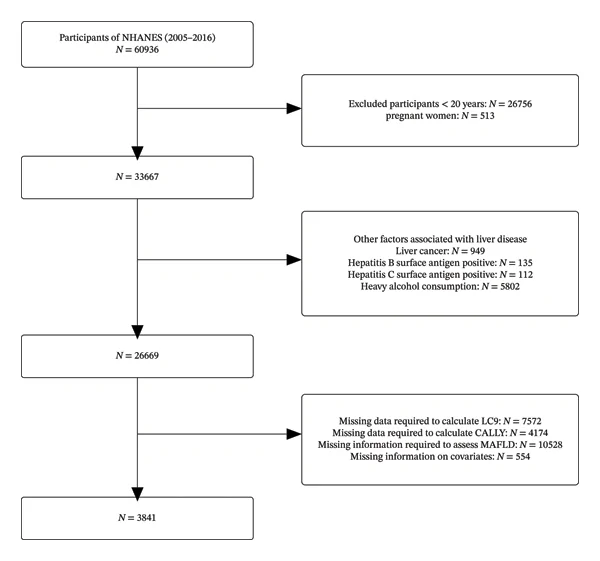
Flowchart for inclusion of study participants from NHANES.

### 2.2. Diagnosis of MAFLD

The diagnosis of hepatic steatosis primarily relies on ultrasound and magnetic resonance imaging (MRI), with liver biopsy performed when necessary for definitive confirmation. However, these imaging techniques are limited by operator dependency, high cost, and reduced sensitivity when hepatic fat content is below 20%–30%, limiting their widespread application. To address these limitations, C.E. Ruhl developed US‐FLI, a tool specifically designed to assess hepatic steatosis in the U.S. population. Previous studies have shown that a US‐FLI score of 30 or higher can be used as a diagnostic threshold for hepatic steatosis [18]. According to the international consensus criteria, MAFLD was defined as the presence of hepatic steatosis together with at least one of the following conditions: overweight/obesity, type 2 diabetes mellitus, or metabolic dysregulation [1].

### 2.3. Definition of LC9 Score

Building on the AHA Life’s Essential 8 (LE8) CVH framework, LC9 has been proposed as an expanded CVH metric that incorporates psychological health as an additional component. The LC9 score comprises the following nine components: physical activity, nicotine exposure, dietary patterns, sleep duration, body mass index (BMI), blood pressure, blood glucose, blood lipids, and psychological health. Psychological health is assessed using PHQ‐9, a widely used instrument for assessing depressive symptoms. Similar to the scoring method of Life’s Essential 8 (LE8), the LC9 score is calculated on a continuous scale ranging from 0 to 100, with each component contributing equally. Each component is standardized to a score between 0 and 100 based on predefined thresholds, and the overall LC9 score is derived as the arithmetic mean of the nine individual component scores [7]. Detailed definitions and scoring criteria for the nine LC9 components are provided in Supporting Table S1.

### 2.4. Definition of CALLY Index

The CALLY index is calculated using the following formula: CALLY = serum albumin level (g/dL) × lymphocyte count (1000 cells/mL)/C‐reactive protein level (mg/dL)/10 [19]. Because the CALLY index exhibited a markedly right‐skewed distribution, a natural logarithmic transformation was applied before analysis. The transformed value is referred to as ln(CALLY) throughout the manuscript.

### 2.5. Covariates

In our study, we included covariates that may have population confounding factors, including age (20–40, 41–60, > 60 years), race (Mexican American, non‐Hispanic White, non‐Hispanic Black, other races), gender (male, female), poverty–income ratio (PIR < 1.3, 1.3–3.5, > 3.5), marital status (married/cohabiting, divorced/separated/widowed, and never married), education level (below high school, high school and above), and alcohol consumption (never, light, moderate, heavy). Laboratory measurements included aspartate transaminase (AST), alanine transaminase (ALT), alkaline phosphatase (ALP), and gamma‐glutamyl transferase (GGT). These biochemical assessments were conducted using standard laboratory methods. Additionally, covariates obtained from questionnaires included diabetes (yes/no), hypertension (yes/no), and hyperlipidemia (yes/no) [20]. Detailed definitions of covariates are provided in Supporting Table S2.

### 2.6. Statistical Analyses

This study followed the NHANES analytic guidelines, and during the data analysis, we considered the complex sampling design and sampling weights. The weight calculation formula was: Weight = 1/6 ∗ WTSAF2YR (WTSAF2YR is the 2‐year subsample weight for each survey period). In descriptive analyses, continuous variables for participant characteristics were expressed as means and standard deviations, while categorical variables were presented as percentages or frequencies. To assess individual differences between the MAFLD and non‐MAFLD groups, chi‐square tests were used for categorical variables, and *t*‐tests and Mann–Whitney U tests were used for continuous variables. Weighted logistic regression was used to assess the associations of LC9 scores and ln(CALLY) with MAFLD, establishing three models. Model 1 was unadjusted for any confounders; Model 2 was adjusted for age, sex, race/ethnicity, marital status, education level, PIR, and alcohol consumption; and Model 3 was further adjusted for hypertension, diabetes, hyperlipidemia, ALT, AST, GGT, and ALP. The relationship between LC9 levels and MAFLD was examined using restricted cubic spline (RCS) regression. To explore the effects in different populations, subgroup analyses were conducted, and potential heterogeneity between subgroups was investigated through interaction terms. Weighted quantile sum (WQS) analysis was performed by calculating weighted linear indices and assigning the corresponding weights to assess the relative contributions of individual LC9 components to the overall association with MAFLD. Furthermore, exploratory mediation analysis was performed using the “mediation” package in R software to quantify the statistical indirect association between LC9 and MAFLD through ln(CALLY). Because of the cross‐sectional design of NHANES, the temporal ordering of LC9, ln(CALLY), and MAFLD could not be established; therefore, the mediation analysis was interpreted as exploratory statistical mediation rather than evidence of a causal pathway. Nonparametric bootstrap resampling with 1000 iterations was used to estimate 95% confidence intervals for the indirect effect, direct effect, total effect, and proportion mediated. Additionally, a series of sensitivity analyses were conducted to ensure the robustness of the results. First, to address the potential definitional overlap between LC9 and the MAFLD diagnostic framework, a modified LC9 score was constructed by excluding BMI, blood glucose, blood pressure, and blood lipid components. The modified LC9 score was calculated as the arithmetic mean of the remaining five components: psychological health, diet, physical activity, nicotine exposure, and sleep health. Second, the prognostic nutritional index (PNI), a CRP‐free immunonutritional marker, was evaluated as an alternative mediator using the same exploratory mediation framework. PNI was calculated as 10 × serum albumin (g/dL) + 0.005 × lymphocyte count (/mm^3^). Third, MASLD was used as an alternative outcome definition. MASLD was defined as hepatic steatosis based on a US‐FLI score ≥ 30 together with at least one cardiometabolic risk factor, after excluding other specific causes of liver disease and alcohol‐related liver disease. The associations of LC9 and ln(CALLY) with MASLD were then evaluated using the same weighted regression framework as in the primary analysis. In addition, to reduce the impact of missing variables on the results, multiple imputations were performed using chained equations to impute missing values, generating five imputed datasets based on the variables in the final statistical model. Weighted logistic regression was also used to assess the associations of LE8 and LS7 with MAFLD, and the results were compared with those for LC9. The associations between individual LC9 components and MAFLD were further evaluated. Finally, FLI‐defined hepatic steatosis was used as an alternative definition to reassess the association between LC9 and MAFLD and the exploratory statistical mediation through ln(CALLY).

## 3. Results

### 3.1. Characteristics of the Participants

A total of 3841 participants were enrolled in this study, of whom 1277 were diagnosed with MAFLD, resulting in a weighted prevalence of 36.7%. Compared to individuals without MAFLD, those with the disease were more likely to be male, middle‐aged, or older, and of Mexican‐American descent. They also had lower levels of income, education, and alcohol consumption. Regarding comorbidities, the prevalence of hypertension, diabetes, and dyslipidemia was significantly higher in the MAFLD group. Biochemically, participants with MAFLD exhibited elevated levels of alanine aminotransferase (ALT), aspartate aminotransferase (AST), GGT, and ALP, along with reduced levels of LC9 and ln(CALLY). Notably, a larger proportion of individuals with MAFLD were concentrated in the lowest tertiles of both LC9 and ln(CALLY) (Table 1).

**TABLE 1:** Weighted baseline characteristics of participants stratified by MAFLD status.

Variables	Total	Non‐MAFLD	MAFLD	*p* value
No. of participants in the sample	3841	2564	1277	
Age				< 0.01
20–40	1068 (30.68)	830 (35.15)	238 (20.80)	
41‐60	1342 (43.28)	881 (42.08)	461 (45.92)	
> 60	1431 (26.05)	853 (22.77)	578 (33.28)	
Sex				< 0.001
Female	2006 (53.07)	1458 (58.33)	548 (41.47)	
Male	1835 (46.93)	1106 (41.67)	729 (58.53)	
Race				< 0.001
Mexican American	592 (6.28)	306 (5.13)	286 (8.83)	
Non‐Hispanic Black	738 (10.04)	597 (11.89)	141 (5.96)	
Non‐Hispanic White	2080 (74.82)	1361 (73.12)	719 (78.57)	
Other	431 (8.86)	300 (9.86)	131 (6.65)	
PIR				0.030
< 1.3	985 (15.64)	597 (14.58)	388 (17.98)	
1.3–3.5	1498 (36.25)	1007 (35.94)	491 (36.94)	
> 3.5	1358 (48.11)	960 (49.48)	398 (45.08)	
Marital status				0.090
No	1387 (30.96)	959 (32.05)	428 (28.55)	
Yes	2454 (69.04)	1605 (67.95)	849 (71.45)	
Education				< 0.001
Below high school	390 (5.09)	196 (4.07)	194 (7.35)	
High school or above	3451 (94.91)	2368 (95.93)	1083 (92.65)	
Alcohol				< 0.001
Former	943 (20.12)	569 (17.83)	374 (25.18)	
Mild	1620 (46.04)	1108 (46.74)	512 (44.48)	
Moderate	687 (20.73)	496 (22.33)	191 (17.18)	
Never	591 (13.12)	391 (13.10)	200 (13.16)	
Hypertension				< 0.001
No	2134 (61.48)	1639 (70.73)	495 (41.04)	
Yes	1707 (38.52)	925 (29.27)	782 (58.96)	
Diabetes				< 0.001
No	3080 (85.30)	2241 (91.77)	839 (71.01)	
Yes	761 (14.70)	323 (8.23)	438 (28.99)	
Hyperlipidemia				< 0.001
No	936 (25.18)	785 (31.46)	151 (11.30)	
Yes	2905 (74.82)	1779 (68.54)	1126 (88.70)	
ALT	24.66 (0.22)	21.86 (0.24)	30.85 (0.61)	< 0.001
AST	24.90 (0.26)	23.87 (0.27)	27.18 (0.48)	< 0.001
GGT	25.10 (0.39)	19.57 (0.28)	37.33 (1.06)	< 0.001
ALP	67.16 (0.53)	64.60 (0.52)	72.83 (0.87)	< 0.001
LC9	71.27 (0.40)	75.00 (0.35)	63.04 (0.48)	< 0.001
LC9, Tertile				< 0.001
T1	1288 (28.49)	606 (18.51)	682 (50.56)	
T2	1278 (32.56)	835 (30.68)	443 (36.73)	
T3	1275 (38.95)	1123 (50.82)	152 (12.72)	
CALLY	6.13 (0.03)	6.38 (0.03)	5.57 (0.04)	< 0.001
CALLY, Tertile				< 0.001
T1	1281 (30.72)	677 (23.50)	604 (46.68)	
T2	1283 (32.98)	845 (32.34)	438 (34.41)	
T3	1277 (36.30)	1042 (44.16)	235 (18.91)	

*Note:* Mean (SD) for continuous variables: the *p* value was calculated by the weighted Student’s *T*‐test. Percentages (weighted *N*, %) for categorical variables: the *p* value was calculated by the weighted chi‐square test. MAFLD, metabolic dysfunction–associated fatty liver disease; CALLY, C‐reactive protein–albumin–lymphocyte index.

Abbreviations: LC9, Life’s Crucial 9; PIR, poverty–income ratio.

### 3.2. Associations of LC9 and ln(CALLY) With MAFLD

Table 2 presents the associations between LC9 scores and ln(CALLY) and the odds of MAFLD. The results show that LC9 scores are significantly and inversely associated with MAFLD, both as a continuous variable and across tertiles. In the fully adjusted Model 3, each 10‐point increase in LC9 was associated with lower odds of MAFLD (OR = 0.51, 95% CI: 0.47–0.56, *p* < 0.001). In the tertile analysis of Model 3, compared with the lowest tertile (T1), participants in the highest tertile (T3) had lower odds of MAFLD (OR = 0.16, 95% CI: 0.12–0.22, *p* < 0.001). Furthermore, RCS analysis revealed a nonlinear inverse association between the LC9 scores and MAFLD (Figure 2A). Similarly, ln(CALLY) was significantly and inversely associated with MAFLD. In the fully adjusted Model 3, higher ln(CALLY) was associated with lower odds of MAFLD (OR = 0.57, 95% CI: 0.52–0.64, *p* < 0.001). Compared with participants in the lowest tertile (T1), those in the highest tertile of ln(CALLY) (T3) had lower odds of MAFLD (OR = 0.21, 95% CI: 0.15–0.28, *p* < 0.001). RCS analysis further demonstrated a nonlinear inverse association between ln(CALLY) and MAFLD (Figure 2B).

**TABLE 2:** Associations of LC9 scores and ln(CALLY) with MAFLD.

Characteristics	Model 1	*p* value	Model 2	*p* value	Model 3	*p* value
LC9‐MAFLD						
Continuous (per 10 scores)	0.44 (0.42, 0.47)	< 0.001	0.41 (0.38, 0.44)	< 0.001	0.51 (0.47, 0.56)	< 0.001
Tertile						
T1	Ref		Ref		Ref	
T2	0.44 (0.36, 0.52)	< 0.001	0.40 (0.33, 0.50)	< 0.001	0.55 (0.44, 0.68)	< 0.001
T3	0.09 (0.07, 0.12)	< 0.001	0.08 (0.06, 0.11)	< 0.001	0.16 (0.12, 0.22)	< 0.001
*p* for trend	< 0.001		< 0.001		< 0.001	
ln(CALLY)‐MAFLD						
Continuous	0.60 (0.56, 0.64)	< 0.001	0.55 (0.51, 0.60)	< 0.001	0.57 (0.52, 0.64)	< 0.001
Tertile						
T1	Ref		Ref		Ref	
T2	0.54 (0.43, 0.66)	< 0.001	0.43 (0.34, 0.55)	< 0.001	0.44 (0.33, 0.57)	< 0.001
T3	0.22 (0.17, 0.28)	< 0.001	0.18 (0.13, 0.23)	< 0.001	0.21 (0.15, 0.28)	< 0.001
*p* for trend	< 0.001		< 0.001		< 0.001	

**FIGURE 2:**
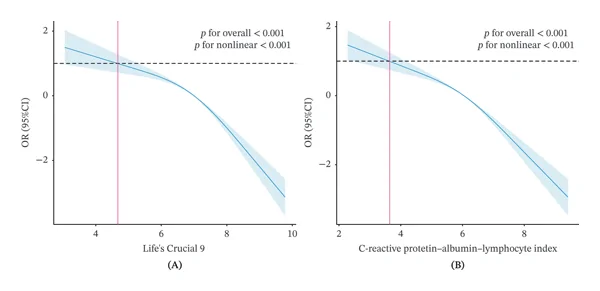
Restricted cubic spline analyses of the associations between LC9 and MAFLD (A) and between ln(CALLY) and MAFLD (B).

### 3.3. Associations of LC9 and ln(CALLY) With MAFLD Across Subgroups

We conducted stratified weighted multivariable regression analyses to examine the associations of LC9 score and the ln(CALLY) index with MAFLD across subgroups defined by sex, age, race, education level, marital status, PIR, hypertension, diabetes, and hyperlipidemia. An inverse association between LC9 score and MAFLD was observed in nearly all subgroups, although the association was not statistically significant among participants with lower education levels. Significant interactions were identified for age, race, education level, and hypertension (all *p* for interaction < 0.05), whereas no significant interactions were observed for sex, PIR, marital status, diabetes, or hyperlipidemia (all *p* for interaction > 0.05) (Figure 3A). Similarly, the ln(CALLY) index showed a consistent inverse association with MAFLD across all subgroups. Significant interactions were observed for race, education level, hypertension, and diabetes (all *p* for interaction < 0.05), whereas no significant interactions were found for age, sex, PIR, marital status, or hyperlipidemia (all *p* for interaction > 0.05) (Figure 3B).

**FIGURE 3:**
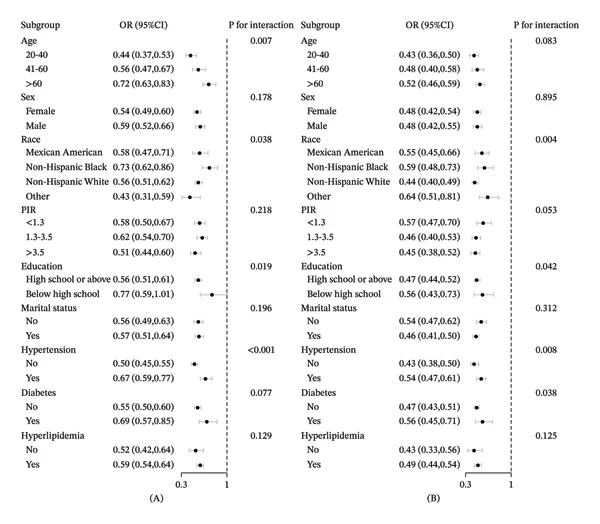
Subgroup analyses of the associations of LC9 and ln(CALLY) with MAFLD. (A) LC9 and MAFLD. (B) ln(CALLY) and MAFLD. Analyses were adjusted for age, sex, education level, marital status, PIR, race, alcohol consumption, hypertension, diabetes, and hyperlipidemia.

### 3.4. WQS Analysis

To evaluate the contribution of individual components within the LC9 scores to the outcome, we conducted a WQS regression analysis. In the WQS analysis, BMI, blood glucose, and psychological health received the largest relative weights among the LC9 components (Figure 4).

**FIGURE 4:**
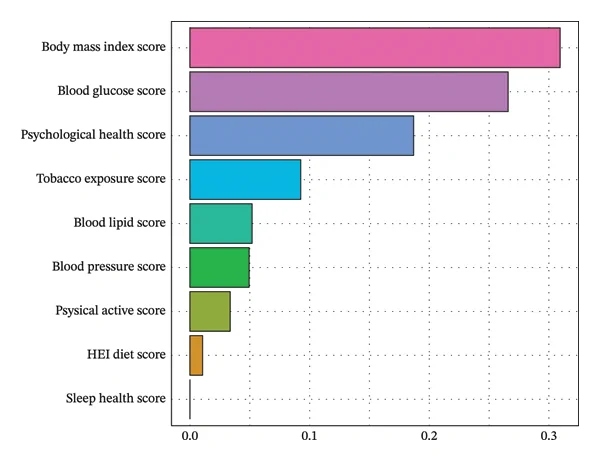
Weights represent the proportion of partial effect for each LC9 metric in the WQS regression. The model adjusted for age, gender, education level, marital, PIR, race, alcohol, hypertension, diabetes, and hyperlipidemia.

### 3.5. Exploratory Mediation Analysis

To further characterize the association between LC9 and MAFLD, we performed an exploratory mediation analysis using ln(CALLY) as the mediator. Both the statistical indirect and direct associations were significant. The average proportion mediated through ln(CALLY) was approximately 17.7% (*p* < 0.001) (Table 3). Given the cross‐sectional design of NHANES, these findings should be interpreted as exploratory statistical mediation and do not establish a causal or temporal pathway linking LC9, ln(CALLY), and MAFLD.

**TABLE 3:** Exploratory mediation analysis of ln(CALLY) in the association between LC9 and MAFLD.

Mediator		Direct effect	Indirect effect (mediated)	Total effect	Proportion mediated (%)
CALLY	Estimate	−0.035	−0.008	−0.043	17.7
	95% CI	(−0.041, −0.027)	(−0.011, −0.005)	(−0.052, −0.032)	
	*p* value	< 0.001	< 0.001	< 0.001	< 0.001

### 3.6. Sensitivity Analysis

To verify the robustness of our findings, we performed multiple imputations. In the fully adjusted Model 3, LC9 remained inversely associated with MAFLD (OR = 0.54, 95% CI: 0.50–0.59, *p* < 0.001) (Supporting Table S4). Similarly, when hepatic steatosis was defined using FLI, LC9 was still significantly inversely associated with MAFLD (OR = 0.40, 95% CI: 0.37–0.44, *p* < 0.001) (Supporting Table S5). Exploratory mediation analysis showed that ln(CALLY) accounted for 16.9% of the statistical association between LC9 and FLI‐defined MAFLD (Supporting Table S8). In Model 3, each 10‐point increase in LE8 was associated with lower odds of MAFLD (OR = 0.54, 95% CI: 0.50–0.57, *p* < 0.001) (Supporting Table S6), while each 10‐point increase in LS7 was also associated with lower odds of MAFLD (OR = 0.63, 95% CI: 0.59–0.68, *p* < 0.001) (Supporting Table S8), with associations broadly consistent with those observed for LC9. In additional exploratory mediation analyses, ln(CALLY) accounted for 17.6% of the association between LE8 and MAFLD and 15.5% of the association between LS7 and MAFLD (Supporting Tables S9 and S10). In analyses of the individual LC9 components, higher diet, BMI, blood lipid, blood glucose, and blood pressure scores were associated with lower odds of MAFLD, whereas psychological health, physical activity, tobacco exposure, and sleep health were not statistically significant in the fully adjusted model (Supporting Table S3).

Additional sensitivity analyses were performed to address potential definitional overlap and alternative outcome classification. After excluding BMI, blood glucose, blood pressure, and blood lipid components from LC9, the association was attenuated but remained statistically significant; each 10‐point increase in the modified LC9 score was associated with lower odds of MAFLD (OR = 0.891, 95% CI: 0.832–0.955, *p* = 0.002) (Supporting Table S11). PNI, a CRP‐free alternative immunonutritional mediator, showed no significant statistical indirect association (*p* = 0.988), with a mediated proportion of only 0.008% (Supporting Table S12). Finally, when MASLD was used as an alternative outcome definition, the inverse association of LC9 remained materially consistent (OR per 10‐point increase = 0.545, 95% CI: 0.494–0.602, *p* < 0.001) (Supporting Table S13).

## 4. Discussion

This study used data from the NHANES conducted between 2005 and 2016 to investigate the association between LC9 scores and prevalent MAFLD and to explore the statistical role of the CALLY index in this association. Higher LC9 scores were significantly associated with lower odds of MAFLD, with each 10‐point increment in LC9 corresponding to an OR of 0.51 (95% CI: 0.47–0.56). Exploratory mediation analysis suggested that ln(CALLY) statistically accounted for approximately 17.7% of the association between LC9 and MAFLD. However, given the cross‐sectional design, this finding should not be interpreted as evidence of a causal or temporal mediation pathway. Importantly, the association was substantially attenuated but remained statistically significant after excluding BMI, blood glucose, blood pressure, and blood lipid components from LC9, suggesting that definitional overlap contributed to the magnitude of the primary association but did not fully explain it. In addition, the inverse association remained materially consistent when MASLD was used as an alternative outcome definition.

The associations of LS7 and LE8 scores with MAFLD have been extensively documented, and the current findings align with previous research indicating a reduced likelihood of MAFLD among individuals exhibiting better CVH metrics. For example, a cross‐sectional study involving 3901 participants from the NHANES database reported an inverse association between LS7 scores and NAFLD prevalence [8]. A cohort study conducted in Korea further supported this finding, suggesting that higher LS7 scores may facilitate the resolution of NAFLD [21]. Another cross‐sectional investigation demonstrated that higher LE8 scores were associated with a lower prevalence of both MAFLD and advanced liver fibrosis [22]. Additionally, a large prospective cohort study conducted in the United Kingdom with a median follow‐up duration of 11.9 years indicated that adherence to healthy lifestyle practices and higher LE8 scores were significantly associated with a reduced risk of incident severe NAFLD, independently of genetic susceptibility [23]. Although previous evidence preliminarily indicated that higher LC9 scores were associated with a decreased risk of NAFLD and reduced all‐cause mortality [11], relevant studies remain limited. Our findings extend this literature by examining LC9 in relation to MAFLD and by evaluating whether the association persists after accounting for potential overlap between LC9 components and the MAFLD diagnostic framework.

An important consideration is that several components of LC9, including BMI, blood glucose, blood pressure, and blood lipids, overlap conceptually with the metabolic abnormalities used in the MAFLD diagnostic framework [1]. Therefore, part of the strong association observed for the full LC9 score may reflect this definitional overlap. In the individual‐component analyses, higher diet, BMI, blood lipid, blood glucose, and blood pressure scores were independently associated with lower odds of MAFLD, whereas psychological health, physical activity, tobacco exposure, and sleep health were not statistically significant in the fully adjusted model. Consistently, after BMI, blood glucose, blood pressure, and blood lipid components were removed, the association of the modified LC9 score with MAFLD was markedly attenuated but remained significant (OR per 10‐point increase = 0.891, 95% CI: 0.832–0.955). These findings indicate that overlapping metabolic components contribute substantially to the magnitude of the association but do not entirely account for it. Nevertheless, because the modified LC9 score has not been externally validated, it should be regarded only as a sensitivity construct rather than an alternative CVH metric.

Individual cardiovascular factors comprising the LC9 score may affect MAFLD development through mechanisms including reducing systemic inflammation, enhancing insulin sensitivity, and mitigating hepatic fat accumulation. After adjusting for confounders, WQS analysis assigned relatively larger weights to BMI, blood glucose, and psychological health. Obesity induces chronic low‐grade inflammation, hormonal dysregulation, and immune imbalance, ultimately resulting in systemic metabolic disturbances that elevate the risk of liver disease [24]. Weight loss is associated with improvements in hepatic steatosis, lobular inflammation, and hepatocellular ballooning degeneration in patients with MAFLD [25]. Hyperglycemia and increased influx of free fatty acids contribute to de novo lipogenesis, enhance hepatic lipid oxidation, and stimulate oxidative phosphorylation, potentially aggravating hepatic oxidative stress, inflammation, and fibrosis [26]. These mechanisms provide biological plausibility for the observed associations, although the present cross‐sectional study cannot establish these mechanistic pathways.

Elevated cortisol promotes gluconeogenesis, impairs insulin‐mediated glucose uptake in peripheral tissues, and increases circulating free fatty acids, ultimately culminating in insulin resistance [27]. The close association between hepatic steatosis and insulin resistance thus provides a plausible mechanistic link between depression and MAFLD. Additionally, depression is associated with a sustained inflammatory state characterized by elevated inflammatory markers, such as CRP and various cytokines, which may further exacerbate hepatic injury [28].

Although previous studies have reported an association between depression and MAFLD [10], psychological health was not independently associated with MAFLD in our fully adjusted individual‐component analysis. In contrast, WQS analysis assigned a relatively large weight to psychological health, suggesting that its contribution may depend on its correlation with other LC9 components rather than an independent association. This discrepancy may reflect the cross‐sectional design, correlations among LC9 components, residual confounding, or limitations of the PHQ‐9 in capturing the broader construct of psychological health. Depression may nevertheless influence several other LC9 domains, including diet, physical activity, smoking, sleep, and body weight [29, 30], and may also be accompanied by neurohumoral activation and systemic inflammatory and metabolic disturbances [31]. Therefore, the contribution of psychological health to the LC9–MAFLD association should be interpreted cautiously, and further longitudinal studies using broader and more objective measures of psychological health are warranted.

The CALLY index is a composite biomarker that integrates markers of inflammation, immunity, and nutritional status, derived from CRP, peripheral blood lymphocyte count, and serum albumin levels. It has previously been used to evaluate prognosis in various cancer populations, including those with gastric cancer, colorectal liver metastases, and non‐small‐cell lung cancer [32–34]. CRP, an acute‐phase protein synthesized by the liver, plays a pivotal role in activating the NF‐κB signaling pathway and promoting inflammatory responses. It also interferes with insulin signaling, thereby contributing to the development and progression of NAFLD [35]. Immune cells, particularly lymphocytes, are key mediators in the pathogenesis of MAFLD. Excessive hepatic lipid accumulation recruits immune cells to the liver, where they participate in lipid‐induced inflammatory responses [36]. Serum albumin reflects nutritional and inflammatory status [13]. A recent multicohort study using NHANES, UK Biobank, and a Chinese clinical cohort reported a consistent inverse association between ln‐CALLY and prevalent MAFLD, providing independent support for our finding that higher ln(CALLY) is associated with lower odds of MAFLD [37]. Our study further extends these observations by exploring the statistical indirect association between LC9 and MAFLD through ln(CALLY).

Nevertheless, the mediation findings require cautious interpretation. Although exploratory mediation analysis identified a statistically significant indirect association through ln(CALLY), temporal ordering among LC9, CALLY, and MAFLD cannot be established in cross‐sectional NHANES data. Moreover, CRP is one of the metabolic abnormalities that may contribute to the definition of metabolic dysregulation under the MAFLD framework, particularly among lean or normal‐weight individuals [1], creating additional conceptual overlap between the mediator and outcome. To examine whether the observed indirect association extended to another immunonutritional marker without CRP, we evaluated PNI as an alternative mediator. PNI has previously been investigated in relation to fatty liver disease in NHANES populations [38]. However, in our study, no significant indirect association was observed through PNI (*p* = 0.988), with an estimated mediated proportion of only 0.008%. Thus, the indirect association observed for ln(CALLY) appears to be marker‐specific and should be regarded as hypothesis‐generating rather than evidence of a general inflammatory or nutritional causal pathway.

The nomenclature of metabolic fatty liver disease has also evolved. In 2023, a multisociety Delphi consensus introduced the term metabolic dysfunction–associated steatotic liver disease (MASLD), defined by hepatic steatosis in the presence of at least one cardiometabolic risk factor, and this terminology has subsequently become increasingly adopted [39]. We retained MAFLD as the primary outcome because the study was originally designed within the MAFLD framework and because MAFLD and MASLD represent related but nonidentical diagnostic constructs. To determine whether our principal finding depended on the disease definition, we additionally repeated the analysis using MASLD. The inverse association remained materially consistent (OR per 10‐point increase in LC9 = 0.545, 95% CI: 0.494–0.602), supporting the robustness of the association across the two diagnostic frameworks. Recent comparative studies likewise indicate substantial, although incomplete, overlap between MAFLD and MASLD populations [40].

Taken together, these findings suggest that higher LC9 scores are associated with a more favorable metabolic liver phenotype and that ln(CALLY) may capture part of the inflammatory, immune, and nutritional variation accompanying this association. However, these findings do not establish that higher LC9 scores or changes in CALLY causally influence the development of MAFLD. The present data are also insufficient to support the use of CALLY as a clinical screening or intervention target. Prospective studies are needed to determine the temporal relationships among LC9, CALLY, and liver health and to evaluate whether CALLY provides incremental value for risk stratification.

This study has several limitations. First, because NHANES is cross‐sectional, the temporal sequence among LC9, ln(CALLY), and MAFLD cannot be established, and reverse causation cannot be excluded. Accordingly, the mediation analyses should be interpreted as exploratory statistical mediation rather than evidence of causal mechanisms. Second, several LC9 components overlap conceptually with metabolic abnormalities used to define MAFLD, which may inflate the magnitude of the primary association. Although the association remained statistically significant after these components were excluded, the modified LC9 score has not been independently validated. Third, CALLY contains CRP, which overlaps with one of the metabolic dysfunction criteria used in the MAFLD framework, particularly among lean or normal‐weight individuals. Moreover, the absence of a significant indirect association using the CRP‐free PNI suggests that the observed indirect association may be specific to ln(CALLY). Fifth, several behavioral and demographic variables were self‐reported and may therefore be subject to recall or reporting bias. Finally, residual and unmeasured confounding cannot be completely excluded despite multivariable adjustment. Prospective cohort and mechanistic studies are needed to clarify the temporal and biological relationships among CVH, inflammatory–nutritional status, and metabolic liver disease.

## 5. Conclusion

Higher LC9 scores were inversely associated with prevalent MAFLD among U.S. adults. Exploratory mediation analysis suggested that ln(CALLY) may statistically account for part of this association, although the cross‐sectional design precludes causal or temporal interpretation. The attenuation observed after excluding overlapping metabolic components indicates that definitional overlap contributes to the magnitude of the LC9–MAFLD association but does not fully account for it. The overall findings were further supported by sensitivity analyses using alternative outcome definitions and analytical approaches. Prospective studies are warranted to confirm these associations and clarify their temporal and biological relationships.

## Author Contributions

Hanlu Li: formal analysis, investigation, methodology, project administration, software, visualization, writing–original draft. Tianju Li: resources, validation, writing–review and editing.

## Funding

This work was supported by the 2025 Youth Research Project of Chongqing Ninth People’s Hospital (Grant No. 2025YJXM‐QN01) and the Chongqing Public Health Key Specialty Project (Grant No. 2025‐57‐10).

## Ethics Statement

The NHANES protocol was approved by the National Center for Health Statistics Research Ethics Review Board, and written informed consent was obtained from all participants.

## Conflicts of Interest

The authors declare no conflicts of interest.

## Supporting Information

Additional supporting information can be found online in the Supporting Information section.

## Supporting information


**Supporting Information** Supporting Table S1 presents the definition and scoring approach for the Life’s Crucial 9 (LC9) score, including the psychological health, health behavior, and health factor domains. Supporting Table S2 summarizes the definitions of covariates used in this study based on NHANES variables. Supporting Table S3 shows the associations between individual LC9 component scores and MAFLD. Supporting Table S4 presents the associations of LC9 and ln(CALLY) with MAFLD. Supporting Table S5 shows the associations of LC9 and ln(CALLY) with MAFLD defined using FLI. Supporting Table S6 presents the associations of Life’s Essential 8 (LE8) and ln(CALLY) with MAFLD. Supporting Table S7 presents the associations of Life’s Simple 7 (LS7) and ln(CALLY) with MAFLD. Supporting Table S8 presents the exploratory mediation analysis of ln(CALLY) in the association between LC9 and FLI‐defined MAFLD. Supporting Table S9 presents the exploratory mediation analysis of ln(CALLY) in the association between LE8 and MAFLD. Supporting Table S10 presents the exploratory mediation analysis of ln(CALLY) in the association between LS7 and MAFLD. Supporting Table S11 presents the association between the modified LC9 score and MAFLD. Supporting Table S12 presents the exploratory mediation analysis using PNI as an alternative mediator. Supporting Table S13 presents the associations of LC9 and ln(CALLY) with MASLD.

## Data Availability

The data analyzed in this study are publicly available from the National Health and Nutrition Examination Survey (NHANES) conducted by the National Center for Health Statistics, Centers for Disease Control and Prevention. The datasets can be accessed through the NHANES website.
