# Supplementary material for: Inverse Association of the Life’s Crucial 9 Score With MAFLD: An Exploratory Mediation Analysis of the C‐Reactive Protein–Albumin–Lymphocyte (CALLY) Index
Source: Int J Endocrinol. 2026 Oct 17;2026:1340865. doi: 10.1155/ije/1340865 (PMC13676161; doi:10.1155/ije/1340865)
Supplement: Supplementary file 1 [file IJE-2026-1340865-s001.docx]

**Supplementary Material**

**Supplementary Table S1. Definition and scoring approach for the Life’s Crucial 9 score.**

| Domain | CVH Metric | Measurement | Quantification and Scoring of CVH Metric |
| --- | --- | --- | --- |
| Psychological  Health | Depressive symptoms | Depression Screener Questionnaire (DPQ) | **Metric:** Nine-item depression screening instrument PHQ-9. Each symptom item in PHQ-9 is scored on a 4-point scale, from 0 (‘not at all’) to 3 (‘nearly every day’), resulting in a total score of 0 to 27 points.  **Scoring:**  Points Level  100 the score of 0 to 4 points  70 the score of 5 to 9 points  30 the score of 10 to 14 points  0 the score of 15 to 27 points |
| Health Behaviors | Diet | Healthy Eating Index-2015 diet score percentile | Quantiles of DASH-style diet adherence  **Scoring (Population):**  Points Quantile  100 ≥95^th^ percentile (top/ideal diet)  80 75^th^ – 94^th^ percentile  50 50^th^ – 74^th^ percentile  25 25^th^ – 49^th^ percentile  0 1^st^ – 24^th^ percentile (bottom/least ideal quartile) |
|  | Physical activity | Self-reported minutes of moderate or vigorous physical activity per week | **Metric:** Minutes of moderate (or greater) intensity activity per week  **Scoring:**  Points Minutes  100 ≥150  90 120 – 149  80 90 – 119  60 60 – 89  40 30 – 59  20 1 – 29  0 0 |
|  | Nicotine exposure | Self-reported use of cigarettes or inhaled nicotine-delivery system | **Metric:** Combustible tobacco use and inhaled NDS use; or secondhand smoke exposure  **Scoring:**  Points Status  100 Never smoker  75 Former smoker, quit ≥5 yrs  50 Former smoker, quit 1 - <5 yrs  25 Former smoker, quit <1 year, or currently using inhaled NDS  0 Current smoker  Subtract 20 points (unless the score is 0) for living with an active indoor smoker in the home |
|  | Sleep health | Self-reported average hours of sleep per night | **Metric:** Average hours of sleep per night  **Scoring:**  Points Level  100 7 – <9  90 9 – <10  70 6 – <7  40 5 – <6 or ≥10  20 4 – <5  0 <4 |
| Health Factors | Body mass index | Body weight (kg) divided by height squared (m^2^) | **Metric:** Body mass index (kg/m^2^)  **Scoring:**  Points Level  100 <25.0  70 25.0 – 29.9  30 30.0 – 34.9  15 35.0 – 39.9  0 ≥40.0 |
|  | Blood lipids | Plasma total and HDL-cholesterol with the calculation of non-HDL-cholesterol | **Metric:** Non-HDL-cholesterol (mg/dL)  **Scoring:**  Points Level  100 <130  60 130 – 159  40 160 – 189  20 190 – 219  0 ≥220  If the drug-treated level, subtract 20 points |
|  | Blood glucose | Fasting blood glucose or casual hemoglobin A1c | **Metric:** Fasting blood glucose (mg/dL) or Hemoglobin A1c (%)  **Scoring:**  Points Level  100 No history of diabetes and FBG <100 (or HbA1c < 5.7)  60 No diabetes and FBG 100 – 125 (or HbA1c 5.7-6.4) (Pre-diabetes)  40 Diabetes with HbA1c <7.0  30 Diabetes with HbA1c 7.0 – 7.9  20 Diabetes with HbA1c 8.0 – 8.9  10 Diabetes with Hb A1c 9.0 – 9.9  0 Diabetes with HbA1c ≥10.0 |
|  | Blood pressure | Appropriately measured systolic and diastolic blood pressure | **Metric:** Systolic and diastolic blood pressure (mm Hg)  **Scoring:**  Points Level  100 <120/<80 (Optimal)  75 120-129/<80 (Elevated)  50 130-139 or 80-89 (Stage I HTN)  25 140-159 or 90-99  0 ≥160 or ≥100  Subtract 20 points if treated level |

**Supplementary Table S2. Definition of variables involved in this study.**

| Variables | Description in NHANES |
| --- | --- |
| Age | Divided into three groups: 20–40 years old, 41–60  years old, >60 years old |
| Gender | Male and Female |
| Race | Mexican American, Non-Hispanic Black, Non-Hispanic White, Other Race |
| Educational level | Below high school, High School, or above |
| Marital status | Yes: Married/Living with partner |
| PIR | Divided into three groups: <1.3, 1.3–3.5, >3.5 |
| Diabetes | Diabetes was defined as a history of previous diabetes, HbA1c level ≥6.5%, or fasting blood glucose level ≥126 mg/dL |
| Hypertension | The diagnostic criteria consist of self-reported hypertension history, the utilization of antihypertensive medication, a systolic blood pressure (SBP) ≥ 140mmHg, or a diastolic blood pressure (DBP) ≥ 90mmHg |
| Hyperlipidemia | (1) Triglyceride (TG) levels ≥150 mg/dl (1.7 mmol/L);(2) Total cholesterol (TC) levels ≥200 mg/dl (5.18 mmol/L);(3) Low-density lipoprotein (LDL) levels ≥130 mg/dl (3.37 mmol/L);(4) High-density lipoprotein (HDL) levels: Men: <40 mg/dl (1.04 mmol/L); Women: <50 mg/dl (1.30 mmol/L) ;(5) Individuals taking cholesterol-lowering drugs are also considered hyperlipidemia. |
| Alcohol | never (had <12 drinks in lifetime);former (had ≥12 drinks in 1 year and did not drink last year, or did not drink last year but drank ≥12 drinks in lifetime);mild = c(1,2), 1 is for female and 2 is for male;moderate = c(2,3), 2 is for female and 3 is for male; or binge >=2 & binge <5;heavy = c(3,4), 3 is for female and 4 is for male; or binge >=5 |

PIR, Ratio of family income to poverty.

**Supplementary Table S3. Associations between individual LC9 component scores and MAFLD**

| **Characteristics** | **Model 1 [OR (95% CI)]** | ***p-value*** | **Model 2 [OR (95% CI)]** | ***p-value*** | **Model 3 [OR (95% CI)]** | ***p-value*** |
| --- | --- | --- | --- | --- | --- | --- |
| Psychological Health score | 0.92(0.87,0.96) | <0.001 | 0.90(0.85,0.95) | <0.001 | 0.95(0.88,1.01) | 0.118 |
| HEI diet score | 0.92(0.89,0.95) | <0.001 | 0.90(0.87,0.93) | <0.001 | 0.91(0.87,0.94) | <0.001 |
| Physical activity score | 0.96(0.94,0.98) | <0.001 | 0.96(0.94,0.98) | <0.001 | 0.99(0.96,1.01) | 0.252 |
| Tobacco exposure score | 0.98(0.96,1.01) | 0.198 | 0.99(0.96,1.02) | 0.563 | 1.00(0.96,1.03) | 0.884 |
| Sleep health score | 0.96(0.93,0.99) | 0.009 | 0.95(0.92,0.99) | 0.008 | 0.99(0.95,1.03) | 0.527 |
| Body mass index score | 0.66(0.64,0.68) | <0.001 | 0.61(0.59,0.64) | <0.001 | 0.61(0.58,0.65) | <0.001 |
| Blood lipid score | 0.88(0.85,0.90) | <0.001 | 0.89(0.86,0.92) | <0.001 | 0.95(0.91,1.00) | 0.045 |
| Blood glucose score | 0.76(0.73,0.79) | <0.001 | 0.76(0.74,0.79) | <0.001 | 0.84(0.79,0.89) | <0.001 |
| Blood pressure score | 0.83(0.81,0.85) | <0.001 | 0.84(0.82,0.87) | <0.001 | 0.94(0.90,0.97) | 0.001 |

Model 1: no covariates were adjusted.

Model 2: adjusted for age, sex, race/ethnicity, marital status, education level, PIR, and alcohol consumption.

Model 3: further adjusted for hypertension, diabetes, hyperlipidemia, ALT, AST, GGT, and ALP in addition to the covariates included in Model 2.

Abbreviation: LC9, Life's Crucial 9; PIR, poverty income ratio; OR, odds ratio; CI, confidence interval.

**Supplementary Table S4. Associations of LC9 and ln(CALLY) with MAFLD after multiple imputation**

| characteristics | MODEL1 | P-value | MODEL2 | P-value | MODEL3 | P-value |
| --- | --- | --- | --- | --- | --- | --- |
| LC9-MAFLD |  |  |  |  |  |  |
| Continuous(per 10 scores) | 0.50 (0.47, 0.53) | <0.001 | 0.45 (0.42, 0.48) | <0.001 | 0.54 (0.50, 0.59) | <0.001 |
| Tertile |  |  |  |  |  |  |
| T1 | Ref |  | Ref |  | Ref |  |
| T2 | 0.45(0.39,0.53) | <0.001 | 0.39(0.33,0.46) | <0.001 | 0.52(0.43,0.62) | <0.001 |
| T3 | 0.12(0.10,0.15) | <0.001 | 0.11(0.09,0.13) | <0.001 | 0.19(0.15,0.24) | <0.001 |
| p for trend | <0.001 |  | <0.001 |  | <0.001 |  |
| CALLY-MAFLD |  |  |  |  |  |  |
| Continuous | 0.66(0.63,0.70) | <0.001 | 0.63(0.59,0.67) | <0.001 | 0.64(0.60,0.69) | <0.001 |
| Tertile |  |  |  |  |  |  |
| T1 | Ref |  | Ref |  | Ref |  |
| T2 | 0.60(0.52,0.70) | <0.001 | 0.51(0.43,0.60) | <0.001 | 0.49(0.41,0.59) | <0.001 |
| T3 | 0.27(0.23,0.32) | <0.001 | 0.23(0.19,0.28) | <0.001 | 0.26(0.21,0.32) | <0.001 |
| p for trend | <0.001 |  | <0.001 |  | <0.001 |  |

**Supplementary Table S5. Associations of LC9 and ln(CALLY) with FLI-defined MAFLD**

| characteristics | MODEL1 | P-value | MODEL2 | P-value | MODEL3 | P-value |
| --- | --- | --- | --- | --- | --- | --- |
| LC9-MAFLD |  |  |  |  |  |  |
| Continuous(per 10 scores) | 0.37(0.34,0.39) | <0.001 | 0.34(0.31,0.38) | <0.001 | 0.40(0.37,0.44) | <0.001 |
| Tertile |  |  |  |  |  |  |
| T1 | Ref |  | Ref |  | Ref |  |
| T2 | 0.37(0.31,0.45) | <0.001 | 0.35(0.29,0.43) | <0.001 | 0.44(0.35,0.56) | <0.001 |
| T3 | 0.07(0.06,0.09) | <0.001 | 0.07(0.05,0.09) | <0.001 | 0.11(0.08,0.15) | <0.001 |
| p for trend | <0.001 |  | <0.001 |  | <0.001 |  |
| CALLY-MAFLD |  |  |  |  |  |  |
| Continuous | 0.54(0.50,0.58) | <0.001 | 0.51(0.47,0.55) | <0.001 | 0.53(0.48,0.57) | <0.001 |
| Tertile |  |  |  |  |  |  |
| T1 | Ref |  | Ref |  | Ref |  |
| T2 | 0.45(0.34,0.58) | <0.001 | 0.38(0.29,0.50) | <0.001 | 0.38(0.29,0.50) | <0.001 |
| T3 | 0.17(0.14,0.21) | <0.001 | 0.14(0.11,0.18) | <0.001 | 0.16(0.13,0.21) | <0.001 |
| p for trend | <0.001 |  | <0.001 |  | <0.001 |  |

**Supplementary Table S6. Associations of Life’s Essential 8 (LE8) and ln(CALLY) with MAFLD**

| characteristics | MODEL1 | P-value | MODEL2 | P-value | MODEL3 | P-value |
| --- | --- | --- | --- | --- | --- | --- |
| LE8-MAFLD |  |  |  |  |  |  |
| Continuous(per 10 scores) | 0.47(0.45,0.49) | <0.001 | 0.44(0.41,0.46) | <0.001 | 0.54(0.50,0.57) | <0.001 |
| Tertile |  |  |  |  |  |  |
| T1 | Ref |  | Ref |  | Ref |  |
| T2 | 0.42(0.34,0.51) | <0.001 | 0.39(0.31,0.49) | <0.001 | 0.51(0.40,0.65) | <0.001 |
| T3 | 0.09(0.07,0.12) | <0.001 | 0.08(0.06,0.11) | <0.001 | 0.17(0.12,0.23) | <0.001 |
| p for trend | <0.001 |  | <0.001 |  | <0.001 |  |
| CALLY-MAFLD |  |  |  |  |  |  |
| Continuous | 0.60(0.56,0.64) | <0.001 | 0.56(0.51,0.60) | <0.001 | 0.58(0.52,0.64) | <0.001 |
| Tertile |  |  |  |  |  |  |
| T1 | Ref |  | Ref |  | Ref |  |
| T2 | 0.54(0.44,0.67) | <0.001 | 0.44(0.35,0.55) | <0.001 | 0.44(0.33,0.57) | <0.001 |
| T3 | 0.22(0.17,0.28) | <0.001 | 0.18(0.13,0.23) | <0.001 | 0.21(0.15,0.28) | <0.001 |
| p for trend | <0.001 |  | <0.001 |  | <0.001 |  |

**Supplementary Table S7. Associations of Life’s Simple 7 (LS7) and ln(CALLY) with MAFLD**

| characteristics | MODEL1 | P-value | MODEL2 | P-value | MODEL3 | P-value |
| --- | --- | --- | --- | --- | --- | --- |
| LS7-MAFLD |  |  |  |  |  |  |
| Continuous(per 10 scores) | 0.57(0.55,0.60) | <0.001 | 0.54(0.51,0.57) | <0.001 | 0.63(0.59,0.68) | <0.001 |
| Tertile |  |  |  |  |  |  |
| T1 | Ref |  | Ref |  | Ref |  |
| T2 | 0.30(0.23,0.38) | <0.001 | 0.27(0.20,0.35) | <0.001 | 0.43(0.31,0.61) | <0.001 |
| T3 | 0.04(0.03,0.07) | <0.001 | 0.04(0.03,0.06) | <0.001 | 0.09(0.06,0.15) | <0.001 |
| p for trend | <0.001 |  | <0.001 |  | <0.001 |  |
| CALLY-MAFLD |  |  |  |  |  |  |
| Continuous | 0.58(0.54,0.62) | <0.001 | 0.53(0.49,0.58) | <0.001 | 0.56(0.50,0.62) | <0.001 |
| Tertile |  |  |  |  |  |  |
| T1 | Ref |  | Ref |  | Ref |  |
| T2 | 0.53(0.41,0.69) | <0.001 | 0.42(0.32,0.53) | <0.001 | 0.41(0.32,0.53) | <0.001 |
| T3 | 0.21(0.16,0.27) | <0.001 | 0.17(0.13,0.22) | <0.001 | 0.20(0.14,0.29) | <0.001 |
| p for trend | <0.001 |  | <0.001 |  | <0.001 |  |

**Supplementary Table S8.** **Mediation analysis exploring the role of the CALLY index in the association between LC9 and MAFLD(FLI)**

| **Mediator** | | **Direct effect** | **Indirect effect (Mediated)** | **Total effect** | **Proportion mediated (%)** |
| --- | --- | --- | --- | --- | --- |
| CALLY | Estimate | -0.0035 | -0.0007 | -0.0043 | 16.9% |
|  | 95% CI | (-0.0067,-0.0019) | (-0.0015,-0.0004) | (-0.0081, -0.0023) |  |
|  | P value | <0.001 | <0.001 | <0.001 | <0.001 |

**Supplementary Table S9.** **Mediation analysis exploring the role of the CALLY index in the association between LE8 and MAFLD**

| **Mediator** | | **Direct effect** | **Indirect effect (Mediated)** | **Total effect** | **Proportion mediated (%)** |
| --- | --- | --- | --- | --- | --- |
| CALLY | Estimate | -0.0434 | -0.0093 | -0.0532 | 17.6% |
|  | 95% CI | (-0.050,-0.040) | (-0.011,-0.010) | (-0.060, -0.043) |  |
|  | P value | <0.001 | <0.001 | <0.001 | <0.001 |

**Supplementary Table S10. Mediation analysis exploring the role of the CALLY index in the association between LS7 and MAFLD**

| **Mediator** | | **Direct effect** | **Indirect effect (Mediated)** | **Total effect** | **Proportion mediated (%)** |
| --- | --- | --- | --- | --- | --- |
| CALLY | Estimate | -0.5308 | -0.0971 | -0.6279 | 15.5% |
|  | 95% CI | (-0.5808,-0.4900) | (-0.1117,-0.0800) | (-0.6864, -0.6000) |  |
|  | P value | <0.001 | <0.001 | <0.001 | <0.001 |

**Supplementary Table S11**. **The association between the modified LC9 score and MAFLD**

| characteristics | MODEL1 | P-value | MODEL2 | P-value | MODEL3 | P-value |
| --- | --- | --- | --- | --- | --- | --- |
| Modified LC9-MAFLD |  |  |  |  |  |  |
| Continuous(per 10 scores) | 0.84(0.79,0.89) | <0.001 | 0.82(0.77,0.88) | <0.001 | 0.89(0.83,0.95) | 0.002 |
| Tertile |  |  |  |  |  |  |
| T1 | Ref |  | Ref |  | Ref |  |
| T2 | 0.73(0.60,0.89) | 0.003 | 0.71(0.57,0.89) | 0.004 | 0.78(0.59,1.03) | 0.074 |
| T3 | 0.52(0.41,0.66) | <0.001 | 0.50(0.38,0.64) | <0.001 | 0.68(0.52,0.90) | 0.009 |
| p for trend | <0.001 |  | <0.001 |  | 0.009 |  |

Note: The modified LC9 score was calculated as the mean of psychological health, diet, physical activity, nicotine exposure, and sleep health scores, after excluding BMI, blood glucose, blood pressure, and blood lipid components.

Model 1: no covariates were adjusted.

Model 2: adjusted for age, sex, race/ethnicity, marital status, education level, PIR, and alcohol consumption.

Model 3: further adjusted for hypertension, diabetes, hyperlipidemia, ALT, AST, GGT, and ALP in addition to the covariates included in Model 2.

Abbreviations: LC9, Life’s Crucial 9; MAFLD, metabolic dysfunction-associated fatty liver disease; PIR, poverty income ratio; OR, odds ratio; CI, confidence interval; ALT, alanine aminotransferase; AST, aspartate aminotransferase; GGT, gamma-glutamyl transferase; ALP, alkaline phosphatase.

**Supplementary Table S12.** Exploratory mediation analysis using PNI as a CRP-free alternative mediator in the association between LC9 and MAFLD

| Mediator | | Direct effect | Indirect effect (Mediated) | Total effect | Proportion mediated (%) |
| --- | --- | --- | --- | --- | --- |
| PNI | Estimate | -0.0453 | −0.000004 | -0.0453 | 0.008% |
|  | 95% CI | −0.0536 to −0.0340 | −0.000291 to 0.000261 | −0.0536 to −0.0341 |  |
|  | P value | <0.001 | 0.988 | <0.001 | 0.988 |

Note: PNI was evaluated as a CRP-free immunonutritional alternative mediator using the same exploratory mediation framework. The indirect effect and mediated proportion were not statistically significant. P values reported as 0 by R are presented as <0.001. PNI, prognostic nutritional index; CRP, C-reactive protein; CI, confidence interval.

**Supplementary Table S13.** The associations of LC9 and ln(CALLY) with MASLD

| characteristics | MODEL1 | P-value | MODEL2 | P-value | MODEL3 | P-value |
| --- | --- | --- | --- | --- | --- | --- |
| LC9-MASLD |  |  |  |  |  |  |
| Continuous(per 10 scores) | 0.52(0.48,0.57) | <0.001 | 0.44(0.40,0.49) | <0.001 | 0.55(0.49,0.60) | <0.001 |
| Tertile |  |  |  |  |  |  |
| T1 | Ref |  | Ref |  | Ref |  |
| T2 | 0.51(0.41,0.64) | <0.001 | 0.46(0.35,0.59) | <0.001 | 0.60(0.47,0.76) | <0.001 |
| T3 | 0.12(0.09,0.16) | <0.001 | 0.09(0.07,0.13) | <0.001 | 0.17(0.12,0.25) | <0.001 |
| p for trend | <0.001 |  | <0.001 |  | <0.001 |  |
| ln(CALLY)-MASLD |  |  |  |  |  |  |
| Continuous | 0.64(0.59,0.69) | <0.001 | 0.56(0.50,0.63) | <0.001 | 0.59(0.52,0.68) | <0.001 |
| Tertile |  |  |  |  |  |  |
| T1 | Ref |  | Ref |  | Ref |  |
| T2 | 0.56(0.44,0.72) | <0.001 | 0.44(0.32,0.59) | <0.001 | 0.44(0.31,0.62) | <0.001 |
| T3 | 0.25(0.19,0.34) | <0.001 | 0.18(0.12,0.26) | <0.001 | 0.21(0.15,0.31) | <0.001 |
| p for trend | <0.001 |  | <0.001 |  | <0.001 |  |

Note: MASLD was defined as hepatic steatosis based on a U.S. Fatty Liver Index (US-FLI) score ≥30 together with at least one cardiometabolic risk factor, after excluding other specific causes of liver disease and alcohol-related liver disease. Model 1 was unadjusted. Model 2 was adjusted for age, sex, race/ethnicity, marital status, education level, PIR, and alcohol consumption. Model 3 was additionally adjusted for hypertension, diabetes, hyperlipidemia, ALT, AST, GGT, and ALP. LC9 was modeled per 10-point increase, whereas ln(CALLY) was modeled per 1-unit increase. Abbreviations: LC9, Life’s Crucial 9; MASLD, metabolic dysfunction-associated steatotic liver disease; CALLY, C-reactive protein–albumin–lymphocyte index; PIR, poverty income ratio; OR, odds ratio; CI, confidence interval; ALT, alanine aminotransferase; AST, aspartate aminotransferase; GGT, gamma-glutamyl transferase; ALP, alkaline phosphatase.
